# Beyond Neurotrophins: A Proposed Neurotrophic–Epigenetic Axis Mediated by Non-Coding RNA Networks for *Hericium erinaceus* Bioactives—A Hypothesis-Driven Review

**DOI:** 10.3390/ijms27031269

**Published:** 2026-01-27

**Authors:** Giovanni Luca Cipriano, Ivana Raffaele, Alessia Floramo, Veronica Argento, Deborah Stefania Donato, Chiara Malatino, Serena Silvestro, Giovanni Schepici, Maria Francesca Astorino, Marco Calabrò, Ivan Anchesi

**Affiliations:** 1 IRCCS Centro Neurolesi “Bonino-Pulejo”, Via Provinciale Palermo, Contrada Casazza, 98124 Messina, Italy; ivana.raffaele@irccsme.it (I.R.); alessia.floramo@irccsme.it (A.F.); veronica.argento@irccsme.it (V.A.); deborah.donato@irccsme.it (D.S.D.); chiara.malatino@irccsme.it (C.M.); serena.silvestro@irccsme.it (S.S.); giovanni.schepici@irccsme.it (G.S.); ivan.anchesi@irccsme.it (I.A.); 2 Department of Biomedical and Dental Sciences and Morpho Functional Imaging, University of Messina, 98125 Messina, Italy; mastorino@unime.it (M.F.A.); mcalabro@unime.it (M.C.)

**Keywords:** *Hericium erinaceus*, non-coding RNA, neurotrophins, neurodegeneration, neuroprotection, neurorehabilitation

## Abstract

*Hericium erinaceus* (*H. erinaceus*), a medicinal mushroom, is a source of bioactive compounds with demonstrated neuroprotective potential. This activity is primarily attributed to two distinct classes of compounds: erinacines from the mycelium, which potently induce the synthesis of neurotrophins, protein growth factors essential for neuronal survival and health, and hericenones from the fruiting body, which subsequently appear to enhance or potentiate neurotrophin-activated signaling pathways. Preclinical evidence substantiates their ability to enhance neurotrophin levels, particularly Nerve Growth Factor (NGF) and Brain-Derived Neurotrophic Factor (BDNF), and activate their cognate Trk receptors. Activation of these pathways, including PI3K/AKT/mTOR and MAPK/ERK, converges on transcription factors such as CREB, promoting neuronal survival, neurite outgrowth, and synaptic plasticity. However, the precise molecular mechanisms linking these small molecules to the complex orchestration of neurotrophic gene expression remain incompletely defined. This review synthesizes current knowledge of the neurotrophic pharmacology of *H. erinaceus* bioactives and proposes a novel framework suggesting that non-coding RNAs (ncRNAs) play a key regulatory role. We hypothesize that hericenones and erinacines modulate key transcriptional hubs, such as CREB, Nrf2, and NF-κB, which in turn regulate the expression of specific ncRNAs (e.g., miR-132, miR-146a) known to control neurogenesis, synaptogenesis, oxidative stress, and neuroinflammation. This ncRNA-mediated mechanism may represent an un-explored axis that explains the pleiotropic neuroprotective effects of these compounds. We critically appraise the existing preclinical evidence, identify significant methodological limitations and translational gaps, and propose a structured research roadmap to test these ncRNA-centric hypotheses, aiming to accelerate the rational development of *H. erinaceus*-derived compounds for neurodegenerative diseases.

## 1. Introduction

### A Triad of Neuroprotection: Hericium erinaceus, Neurotrophins, and Non-Coding RNAs

Neurodegenerative diseases, including Alzheimer’s disease (AD), Parkinson’s disease (PD), and amyotrophic lateral sclerosis (ALS), represent a growing global health crisis characterized by the progressive loss of neuronal structure and function [1]. Despite decades of research, current therapeutic strategies remain largely symptomatic. This therapeutic plateau underscores an urgent need for agents capable of engaging complex biological targets [2]. In this context, natural products have emerged as a rich reservoir of chemical diversity, offering unique molecular scaffolds that may overcome the limitations of conventional synthetic drugs [3]. Among natural sources, the culinary-medicinal mushroom *Hericium erinaceus* (Bull.: Fr.) Pers., commonly known as Lion’s Mane, has a long history of use in traditional Asian medicine for various ailments. Over the past two decades, it has gained significant scientific attention for its profound and pleiotropic effects on the nervous system. The neuroprotective effects of *H. erinaceus* are attributed to two classes of bioactive compounds: hericenones, isolated from the fruiting body, and erinacines, found in the mycelium [4]. A critical pharmacological property of these compounds is their demonstrated ability to cross the blood–brain barrier (BBB), a prerequisite for direct action within the central nervous system (CNS) [5,6,7]. This distinguishes them from larger bioactive molecules like polysaccharides and positions them as prime candidates for CNS-targeted therapies. The foundational rationale for the neuroprotective effects of *H. erinaceus* centers on the “neurotrophic hypothesis.” This hypothesis posits that the survival, differentiation, and functional maintenance of neurons depend on a continuous supply of protein growth factors known as neurotrophins [8]. The canonical members of this family are Nerve Growth Factor (NGF) and Brain-Derived Neurotrophic Factor (BDNF) [9,10]. NGF, acting through its high-affinity receptor Tropomyosin receptor kinase A (TrkA), is essential for the survival of basal forebrain cholinergic neurons and peripheral sensory neurons [11]. BDNF, which signals through TrkB, is ubiquitously expressed in the CNS and is a master regulator of synaptic plasticity, learning, and memory [12]. A large body of preclinical work suggests that the primary mechanism of action for *H. erinaceus* bioactives is their ability to stimulate the endogenous synthesis and release of these vital neurotrophins [13]. However, the regulation of gene expression is vastly more complex than a simple DNA-to-protein paradigm. Beyond neurotrophin regulation, gene expression is further modulated by non-coding RNAs (ncRNAs), which play a critical role in various biological processes, including in the nervous system [14]. Once dismissed as “transcriptional junk,” ncRNAs are now recognized as critical players in virtually all biological processes, with particularly high abundance and specific expression patterns in the brain [15]. For the purposes of this review, three major classes are of interest: MicroRNAs (miRNAs), which are short (~22 nucleotide) ncRNAs that function as post-transcriptional repressors by binding to the 3′ untranslated region (3′ UTR) of target messenger RNAs (mRNAs), leading to their degradation or translational inhibition; Long Non-coding RNAs (lncRNAs), a heterogeneous class of transcripts longer than 200 nucleotides, which can act as molecular scaffolds for protein complexes, decoys for transcription factors, or guides for chromatin-modifying enzymes to regulate gene expression epigenetically and transcriptionally [15]; and Circular RNAs (circRNAs), which are formed by a “back-splicing” event, creating a covalently closed loop. This structure confers exceptional stability, making them ideal candidates for acting as long-lasting molecular “sponges” that sequester miRNAs or RNA-binding proteins [16].

While the link between *H. erinaceus* and neurotrophin induction is well-established, the precise molecular steps connecting compound exposure to altered gene expression remain partially elucidated. The current literature lacks a cohesive framework that integrates the mushroom’s pleiotropic effects—spanning neurotrophic, antioxidant, and anti-inflammatory activities—under a unified mechanism. This review aims to bridge this gap. We will first critically synthesize the existing evidence for the modulation of neurotrophin signaling pathways by hericenones and erinacines. Subsequently, we will introduce and develop a series of testable hypotheses proposing that ncRNA networks serve as a pivotal, and thus far overlooked, mechanistic layer that translates the initial signaling events triggered by these compounds into the sustained changes in gene expression required for neuroprotection and neural repair.

## 2. Mechanistic Evidence on Neurotrophins and Downstream Signaling

The neuroprotective activity of *H. erinaceus* is fundamentally linked to its capacity to modulate the expression and signaling of key neurotrophins. To strengthen the scientific rigor of this evidence, it is essential to distinguish between findings consistently replicated across independent laboratories and those currently limited to specific models. Preclinical studies have delineated a pathway beginning with the induction of NGF and BDNF, followed by receptor activation and the engagement of canonical intracellular cascades that ultimately promote neuronal survival and plasticity [13].

The most consistently reported bioactivity of *H. erinaceus* compounds is the stimulation of neurotrophin synthesis, particularly NGF. In vitro studies using cultured astrocytes, the primary source of NGF in the CNS, have provided quantitative evidence for this effect. Multiple erinacines, including A, C, E, F, and H, have been shown to potently induce NGF secretion [17,18,19]. These findings are corroborated in vivo, where oral administration of erinacine A (8 mg/kg) to rats resulted in significantly increased NGF content in the hippocampus and locus coeruleus, brain regions critical for memory and arousal [20]. Evidence for BDNF induction is also emerging. Erinacine C has been shown to induce both NGF and BDNF expression in the 1321N1 human astrocytoma cell line [8]. Furthermore, administration of *H. erinaceus* mycelium extract to mice enhanced the production of BDNF and its precursor, pro-BDNF, in the hippocampus [13,21]. In contrast to the clear effects of erinacines, the role of hericenones is more ambiguous. While often cited as NGF inducers, several primary studies have reported that purified hericenones C, D, and E failed to directly stimulate NGF mRNA expression in cultured glial cells [8]. However, other work demonstrates that hericenone E can potentiate NGF-induced neurite outgrowth in PC12 cells, suggesting it may function as an allosteric modulator or enhancer of NGF signaling rather than a direct inducer of its synthesis [1,22,23]. We propose a functional dichotomy that may resolve these conflicting findings: erinacines likely drive the de novo synthesis of neurotrophic ligands (ligand supply), whereas hericenones function primarily to sensitize the downstream receptor machinery or potentiate signal transduction (signal amplification). This synergistic dual-action model suggests that whole extracts may offer superior efficacy by simultaneously increasing neurotrophin availability and lowering the threshold for receptor activation.

This model could be experimentally validated by using protein synthesis inhibitors (e.g., cycloheximide) to determine if hericenones can enhance Trk signaling even when de novo neurotrophin production is blocked, thereby proving their role as signal amplifiers. Additionally, dose–response matrix studies utilizing purified compounds could quantify the synergistic indices of erinacine-hericenone combinations, providing a mathematical basis for the efficacy of whole extracts.

Once synthesized and released, the biological effects of NGF and BDNF are transduced by their respective high-affinity receptors, TrkA and TrkB. Evidence confirms that *H. erinaceus* compounds engage these receptor systems and their downstream signaling pathways. Studies have shown that the neuritogenic effects of *H. erinaceus* extracts and isolated compounds are dependent on the TrkA/Erk1/2 pathway [13]. For example, the differentiation of PC12 cells induced by conditioned media from erinacine C-treated astrocytes is mediated by TrkA and its downstream effectors [24]. Similarly, mycelial extracts have been shown to induce TrkB activation in vivo, consistent with an increase in BDNF abundance [5].

Downstream of receptor activation, two major signaling cascades are consistently implicated. The first is the Phosphoinositide 3-kinase (PI3K)/AKT pathway, a canonical pro-survival pathway that inhibits apoptosis and promotes cellular growth [25]. Erinacine A-enriched mycelium has been shown to activate the full BDNF/TrkB/PI3K/Akt/GSK-3β signaling axis in mouse models of depression, correlating with antidepressant-like effects [26,27]. Hericenone E also enhances the phosphorylation of Akt, a central kinase in this pathway [28]. Activation of this cascade is a key mechanism by which *H. erinaceus* compounds protect neurons from apoptotic cell death [29]. The second major cascade is the Mitogen-Activated Protein Kinase (MAPK)/Extracellular Signal-Regulated Kinase (ERK) Pathway. This pathway is fundamentally involved in regulating neuronal plasticity, differentiation, and neurite outgrowth [30]. Numerous studies confirm that *H. erinaceus* compounds robustly activate ERK1/2 phosphorylation [17]. This activation appears to be a central convergence point for multiple neurotrophic signals. For instance, recent work has identified a novel pan-neurotrophic pathway activated by other *H. erinaceus* compounds, hericene A and N-de phenylethyl isohericerin (NDPIH) [13]. These molecules promote extensive neurite outgrowth and enhance memory by promoting ERK1/2 signaling through a mechanism that is largely independent of direct TrkB activation [13]. This finding challenges a simple BDNF-mimetic model and suggests that *H. erinaceus* contains a suite of compounds that can engage neurotrophic machinery through parallel and complementary pathways, all converging on ERK1/2.

At the terminus of these cascades lies the transcription factor cAMP response element-binding protein (CREB), a master regulator of genes involved in neuronal plasticity, learning, and memory. The phosphorylation and activation of CREB have been demonstrated following treatment with *H. erinaceus* extracts in animal models of cerebellar ataxia [31] and traumatic brain injury (TBI) [29], and are linked to the activation of downstream targets and improved cognitive outcomes [5]. The convergence of multiple signaling pathways on CREB positions it as a critical transcriptional hub for mediating the long-term effects of *H. erinaceus* bioactives.

## 3. The Regulatory Landscape of Non-Coding RNAs in Neuronal Function and Pathology

To build a hypothesis linking *H. erinaceus* to ncRNA networks, it is essential to first understand the established roles of these regulatory molecules in the CNS. A functional hierarchy exists within this network: while lncRNAs and circRNAs often function as upstream ‘architectural’ regulators or molecular sponges that maintain transcriptomic stability [32], miRNAs act as the final downstream effectors, translating transient signaling pulses into sustained changes in protein expression [33]. In the context of *H. erinaceus*, we propose that long-lived circRNAs and lncRNAs provide the regulatory framework for neural repair, while miRNAs function as dynamic switches to lock neurons into a pro-plasticity state. MiRNAs, lncRNAs, and circRNAs form a complex, interconnected web that fine-tunes gene expression programs underlying neuronal development, plasticity, and the response to pathological insults. Among these, MicroRNAs (miRNAs) are powerful post-transcriptional regulators, and a subset of them are highly enriched in the brain, where they play indispensable roles [34]. Key examples include the miR-132/212 family, often termed “neurimmiRs,” which are central to neuronal plasticity. Their expression is induced by neuronal activity and neurotrophins (e.g., BDNF) via the CREB transcription factor [35], and functionally, miR-132 promotes dendritic growth, arborization, and the formation and maturation of dendritic spines [36]. miR-132 is specifically prioritized in this framework because it represents the direct molecular bridge between CREB activation, a key effect of erinacines, and the structural remodeling of synapses required for neural repair [37]. Another crucial example is miR-124, one of the most abundant miRNAs in the CNS, which acts as a master regulator of neuronal identity by promoting neurogenesis while actively repressing non-neuronal gene programs [38]. It also possesses anti-inflammatory properties by modulating microglial activation. Similarly, miR-9 is a key player in neurogenesis, guiding the differentiation of neural stem cells [39], while miR-146a serves as a critical negative feedback regulator of the innate immune response. Its transcription is induced by NF-κB, and it subsequently targets adapter proteins like TRAF6 and IRAK1 to dampen the inflammatory cascade, making it crucial for controlling microglial activation and preventing chronic neuroinflammation [40,41]. This miRNA is central to our hypothesis as it offers a mechanistic explanation for how *H. erinaceus* bioactives resolve chronic neuroinflammation by normalizing the NF-κB/miR-146a feedback loop in glial cells. In a complementary fashion, some miRNAs act as potent ‘brakes’ on synaptic plasticity. A striking example is miR-134, a brain-specific miRNA localized to the synapto-dendritic compartment. miR-134 actively represses dendritic spine size and maturation by inhibiting the translation of its target, Limk1 mRNA [42]. Notably, this molecular brake is ‘released’ by pro-plasticity stimuli, such as BDNF, which relieves the miR-134-mediated inhibition, thus permitting spine growth [43]. This network also includes Long Non-coding RNAs (lncRNAs), which are architectural regulators. LncRNAs are a diverse class of transcripts that execute their functions by acting as scaffolds, decoys, or guides for epigenetic modifiers [14]. Approximately 40% of all identified lncRNAs are expressed specifically in the brain [15], underscoring their importance. Notable examples include BDNF-AS, a natural antisense lncRNA that negatively regulates BDNF expression by forming an RNA duplex with its mRNA [44,45,46] MALAT1 (Metastasis-Associated Lung Adenocarcinoma Transcript 1), despite its name, is highly expressed in neurons and is involved in regulating alternative splicing, synaptogenesis, and CREB signaling [47,48]. Furthermore, NEAT1 (Nuclear Enriched Abundant Transcript 1) is an essential structural component of nuclear paraspeckles involved in the cellular stress response and has been linked to neuroinflammatory diseases [47]. Finally, Circular RNAs (circRNAs) act as stable sponges and regulators. These molecules are exceptionally stable due to their covalently closed-loop structure, which renders them resistant to exonuclease degradation [49]. This stability, combined with their high abundance in the brain, makes them potent and long-lasting regulators. The archetypal example is CDR1as (Cerebellar Degeneration-Related protein 1 antisense), also known as ciRS-7. This highly conserved circRNA harbors more than 70 binding sites for miR-7 [16,50]. CDR1as is highly relevant due to its exceptional stability in the brain [51], potentially acting as a long-term molecular reservoir that maintains physiological control over pathogenic proteins, such as α-synuclein, which are targeted by *H. erinaceus* compounds in Parkinson’s disease models. By sequestering miR-7, CDR1as effectively de-represses miR-7’s targets, such as α-synuclein (implicated in PD), making the CDR1as/miR-7 axis a critical factor in neurological diseases. Another well-characterized circRNA is circHIPK3, which acts as a sponge for multiple miRNAs, including miR-124, thereby influencing processes such as cell proliferation, apoptosis, and neuronal function [52].

## 4. Bridging the Gap: Hypothesized Links Between *Hericium erinaceus* Bioactives and ncRNA Networks

### 4.1. Mechanistic Hypotheses: Integrating Transcription Factors and ncRNA Networks

No direct experimental evidence currently links *H. erinaceus* bioactives to ncRNA modulation. However, while the activation of upstream cascades (MAPK/ERK, PI3K/Akt) and transcription factors (CREB, Nrf2, NF-κB) is well-supported, the link to specific ncRNA networks remains a speculative, hypothesis-driven model. We propose that ncRNAs act as ‘architectural stabilizers’ that convert transient kinase signals into sustained structural remodeling. This framework positions ncRNAs as the missing link required to maintain the ‘on-state’ of neurogenic programs long after initial compound exposure.

Established Evidence: *H. erinaceus* bioactives, particularly erinacines, are established activators of the MAPK/ERK and PI3K/AKT signaling cascades. These pathways converge on the phosphorylation and activation of the transcription factor CREB, a response demonstrated in multiple models of *H. erinaceus*-mediated neuroprotection [31]. The promoter region of the miR-212/132 locus contains consensus CREB binding sites, and CREB is a direct and potent transcriptional activator of these miRNAs [36]. Functionally, miR-132 is a well-characterized “pro-neurogenic” regulator that promotes dendritic growth, arborization, and spine formation by targeting inhibitors of synaptic remodeling [36]. This hub is further reinforced by evidence that other *H. erinaceus* compounds, such as Hericene A, can activate ERK/CREB signaling and promote plasticity through pathways independent of TrkB [13,53].

Proposed Model: We hypothesize that the neurite outgrowth and synaptic enhancement attributed to *H. erinaceus* are not solely due to direct neurotrophin upregulation but are mediated indirectly through a CREB-dependent induction of miR-132. In this framework, miR-132 acts as a downstream amplifier and architectural stabilizer that converts transient signaling pulses into sustained structural changes. By recruiting this ncRNA, the compounds maintain the “on-state” of neurogenic programs long after the initial stimulus. This positions the CREB/ncRNA axis as a critical molecular bottleneck and a unifying mechanism for the mushroom’s pleiotropic neurotrophic effects.

Furthermore, the potent antioxidant activity of *H. erinaceus* compounds, especially the unique ability of erinacines A and C to activate the Nrf2 pathway, suggests another point of intersection with ncRNA biology Established Evidence: Erinacines A and C are potent activators of the Nrf2 pathway, inducing its nuclear translocation to bind Antioxidant Response Elements (AREs) in the promoters of target genes [29]. Beyond canonical protein-coding enzymes, Nrf2 directly regulates the expression of certain ncRNAs, while specific miRNAs and lncRNAs reciprocally modulate Nrf2 activity, forming a complex feedback and feed-forward “regulon” [14,54,55]. As a proposed model: we hypothesize that the powerful and lasting antioxidant effects of erinacines result from the activation of this entire Nrf2-ncRNA regulon. By upregulating specific antioxidant-associated miRNAs or lncRNAs, erinacines could induce a more resilient and multi-faceted cytoprotective state than direct protein expression alone, providing a mechanistic basis for their superior efficacy in models of severe oxidative stress.

**Figure 1:**
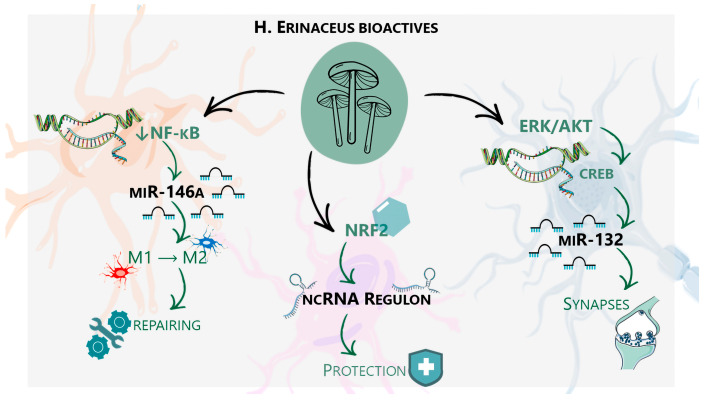
The proposed Neurotrophic-Epigenetic Axis. A schematic representation of the hypothesized mechanisms by which *H. erinaceus* bioactives modulate neuronal and glial function. The framework posits that the compounds engage three master transcriptional hubs (NF-κB, Nrf2, CREB), which in turn regulate specific non-coding RNA networks. (Left) Inhibition of NF-κB upregulates miR-146a, promoting the microglial shift from a pro-inflammatory M1 to a neurorestorative M2 phenotype. (Center) Activation of Nrf2 induces a cytoprotective ncRNA regulon, reducing oxidative stress. (Right) Activation of ERK/AKT signaling phosphorylates CREB, inducing miR-132, a key driver of synaptogenesis and neurite outgrowth.

Finally, as an established evidence, the anti-inflammatory properties of *H. erinaceus* compounds are well-documented, primarily through their inhibition of the pro-inflammatory NF-κB pathway [3]. In glial cells, inflammatory stimuli activate the NF-κB pathway, leading to the transcription of pro-inflammatory cytokines such as TNF-α and IL-1β [56]. The MIR146A gene promoter contains NF-κB binding sites, making it a direct transcriptional target that establishes a crucial negative feedback loop [40]. Induced miR-146a then targets the mRNAs of TRAF6 and IRAK1 to resolve the inflammatory response [57]. As a Proposed Model: We hypothesize that *H. erinaceus* bioactives prevent the pathological over-activation and subsequent exhaustion of the miR-146a feedback system. By dampening the initial pro-inflammatory surge, these compounds “re-sensitize” the innate immune response, shifting microglia and astrocytes from a chronic pro-inflammatory (M1-like) to a homeostatic, neuroprotective phenotype (M2-like). While plausible, these ncRNA-centric axes likely coexist with other mechanisms. The sustained effects of *H. erinaceus* may be driven by direct epigenetic modifications (histone acetylation, DNA methylation), mRNA stabilization via RNA-binding proteins, or the modulation of mitochondrial biogenesis. Acknowledging this interplay is essential for a holistic understanding of these pleiotropic bioactives [58] as depicted in Figure 1.

### 4.2. Constraints and Limitations of the Proposed Framework

While conceptually robust, the proposed neurotrophic-epigenetic axis faces several limitations. First, the link between *H. erinaceus* bioactives and specific ncRNA modulation is currently inferential, based on the activation of shared transcriptional hubs rather than direct ncRNA sequencing data. Second, the inherent complexity of ncRNA networks—where a single miRNA can target hundreds of mRNAs—makes it difficult to isolate the specific contribution of the proposed axes to the observed neuroprotective phenotype. Furthermore, most evidence for *H. erinaceus* bioactivity stems from rodent models, leaving a significant translational gap regarding human pharmacokinetics and long-term safety. Finally, the lack of standardized extraction protocols across the literature complicates the comparison of results and the identification of the exact compounds responsible for ncRNA modulation.

## 5. Disease-Focused Perspectives: Mapping Pathways to Pathologies

The pleiotropic effects of *H. erinaceus* compounds on neurotrophin signaling, oxidative stress, and neuroinflammation suggest their potential utility across a range of neurological disorders. By mapping these mechanisms to the specific pathologies of different diseases, and integrating our ncRNA-centric hypotheses, we can begin to frame a rational basis for their therapeutic application.

In Alzheimer’s disease (AD), pathology is characterized by extracellular amyloid-β plaques, intracellular hyperphosphorylated tau neurofibrillary tangles, and marked synaptic and neuronal loss, especially in the hippocampus and cortex [59]. Preclinical studies in the APPswe/PS1dE9 transgenic mouse model show that erinacine A-enriched mycelial extracts can significantly reduce Aβ plaque burden, increase the expression of insulin-degrading enzyme (IDE), diminish the activation of plaque-associated microglia and astrocytes, and increase the ratio of mature NGF to its precursor, proNGF [60]. These molecular changes correlate with improved performance in behavioral tasks [60]. From an ncRNA perspective, the modulation of Aβ production is a prime area for ncRNA involvement. The lncRNA BACE1-AS is a natural antisense transcript that positively regulates the expression of BACE1, the rate-limiting enzyme for Aβ generation [61]. BACE1-AS expression is known to be upregulated by cellular stressors. Given the potent antioxidant capacity of *H. erinaceus* and its ability to modulate the Nrf2 pathway, we hypothesize that these compounds may indirectly downregulate BACE1-AS by mitigating the upstream oxidative stress that drives its expression. This would represent a restoration of homeostatic ncRNA levels rather than a direct molecular inhibition, a distinction that warrants specific investigation in AD models. Furthermore, numerous miRNAs are known to regulate APP processing and tau phosphorylation [62]. An ncRNA-focused investigation could reveal that the therapeutic effects of these compounds in AD models are mediated by the normalization of a pathogenic ncRNA signature.

In Parkinson’s disease (PD), progressive loss of dopaminergic neurons in the substantia nigra pars compacta and α-synuclein aggregation (Lewy bodies) underlie motor deficits, with microglia-driven neuroinflammation contributing to neuronal degeneration [63]. Erinacine A has demonstrated significant neuroprotective effects in toxin-based PD models (MPTP/MPP+), where it protects dopaminergic neurons from cell death, reduces oxidative stress, and ameliorates motor dysfunction [4]. Crucially, erinacine A also prevents LPS-induced glial cell activation and protects dopaminergic neurons from inflammation-induced death in vitro and in vivo [64]. The ncRNA perspective here is that the regulation of α-synuclein is a key pathogenic node in PD where ncRNAs play a critical role. The circRNA CDR1as acts as a sponge or reservoir for miR-7, which directly targets and suppresses α-synuclein mRNA [50]. Dysregulation of this axis can lead to α-synuclein accumulation. We hypothesize that the neuroprotective effects of *H. erinaceus* in PD models may involve the stabilization or upregulation of the CDR1as/miR-7 axis, thereby maintaining physiological control over α-synuclein levels. Additionally, the compound’s ability to modulate the NF-κB/miR-146a axis could be central to its suppression of microglial-driven neuroinflammation.

In multiple sclerosis (MS) and related demyelinating disorders, autoimmune CNS inflammation targets myelin, causing demyelination, axonal injury, and progressive neurological disability [65]. In this context, therapeutic strategies aim to modulate the immune response and promote remyelination. While clinical data is sparse, preclinical evidence is highly encouraging. In the cuprizone model of demyelination, erinacine S was remarkably effective at preserving oligodendrocytes (the myelin-producing cells), inhibiting demyelination and gliosis, and alleviating associated mood abnormalities [65]. This suggests a direct effect on myelin biology, beyond general anti-inflammatory actions. From an ncRNA perspective, the differentiation of oligodendrocyte precursor cells (OPCs) into mature, myelinating oligodendrocytes is a tightly regulated process governed by specific transcription factors and ncRNAs [66]. It is highly plausible that the potent pro-myelinating effects of erinacine S are mediated by its ability to modulate the expression of miRNAs or lncRNAs that act as key switches in the OPC differentiation program. Identifying these specific ncRNA targets could open new avenues for pro-remyelination therapies.

Finally, in Traumatic Brain Injury (TBI) and Ischemic Stroke, both of which involve an acute primary injury followed by a devastating secondary cascade of excitotoxicity, oxidative stress, and neuroinflammation that expands the lesion and worsens functional outcomes, erinacine A and mycelial extracts have shown significant protective effects. In stroke models, they reduce infarct volume and suppress key inflammatory and cell death pathways, including iNOS and p38 MAPK [67]. In TBI models, *H. erinaceus* mycelium and erinacine C improve functional recovery, reduce neuronal death, and inhibit microglial activation, with effects linked to the activation of the Nrf2 and CREB pathways [29]. The ncRNA perspective suggests that the acute injury environment of TBI and stroke triggers massive and rapid changes in the cellular transcriptome, including widespread dysregulation of ncRNAs involved in apoptosis, inflammation, and cell survival [39,41,50]. The protective effects of *H. erinaceus* compounds in these acute settings are likely due to their ability to rapidly engage powerful cytoprotective pathways (e.g., Nrf2) and anti-inflammatory mechanisms (e.g., NF-κB inhibition) [29,68]. We hypothesize that this involves the normalization of the expression of injury-induced pathogenic ncRNAs and the enhancement of protective ncRNAs, thereby tipping the cellular balance from death and dysfunction toward survival and repair. Our framework distinguishes between conserved “pan-disease” ncRNA responses and disease-specific regulatory nodes. The NF-κB/miR-146a axis and Nrf2-ncRNA regulon serve as universal therapeutic denominators across AD, PD, MS, and acute brain injuries by mitigating neuroinflammation and oxidative stress. Conversely, specialized ncRNAs function as discrete pathological switches: the BACE1-AS/BACE1 axis targets amyloidogenesis in AD, the CDR1as/miR-7 axis regulates α-synuclein dynamics in PD, and myelin-specific ncRNAs govern remyelination programs in MS.

Regarding experimental prioritization, AD and PD models offer high translational readiness given the robust evidence linking erinacines to reduced protein aggregation and glial normalization. However, TBI and ischemic stroke represent the most viable contexts for rapid hypothesis verification; their acute nature permits precise temporal monitoring of the transcriptomic “flip” in neuroprotective ncRNAs, such as miR-132 and miR-146a, following compound exposure. While MS models are promising based on erinacine S data, identifying the specific ncRNA switches governing oligodendrocyte precursor cell (OPC) differentiation remains a prerequisite for large-scale validation.

## 6. Knowledge Gaps and a Framework for Future Research

The synthesis of the current literature reveals a field ripe with potential but constrained by a lack of mechanistic depth and methodological rigor. To move forward, research must pivot from descriptive studies using uncharacterized extracts to hypothesis-driven investigations using standardized compounds and modern molecular tools.

The primary knowledge gap is the absence of a mechanistic link between *H. erinaceus* bioactives and the ncRNA regulatory network. This gap can be addressed by testing a series of specific, falsifiable hypotheses. The prioritization of the described hypotheses is based on their convergence with the three most robustly documented transcriptional hubs—CREB, Nrf2, and NF-κB—implicated in *H. erinaceus* neuroprotection. These nodes represent the most mechanistically plausible intersections where transient signaling cascades are likely translated into long-term epigenetic stability, offering a unified explanation for the mushroom’s pleiotropic effects. (CREB/miR-132) hypothesis posits that the neurotrophic effects of erinacine A are dependent on CREB-mediated upregulation of miR-132 [5,34,35,37]; this would be falsified if pharmacological or genetic inhibition of CREB or miR-132 fails to block erinacine A-induced neurite outgrowth. (Nrf2/ncRNA) hypothesis proposes that the antioxidant effects of erinacines A and C are mediated by an Nrf2-dependent ncRNA regulon [5,29,55,68,69,70]; this is falsified if the antioxidant gene expression profile induced by erinacines in Nrf2-knockout cells is identical to that in wild-type cells, and no Nrf2-dependent ncRNAs are identified via RNA-seq. (NF-κB/miR-146a) hypothesis suggests that the anti-inflammatory effects of *H. erinaceus* compounds involve the normalization of the NF-κB/miR-146a negative feedback loop in glial cells [1,3,5,27,41,64,68,71]; this would be falsified if the compounds inhibit LPS-induced cytokine production to the same extent in wild-type and miR-146a-deficient microglia.

To test these hypotheses, a structured, multi-stage experimental roadmap is proposed. This begins with Tier 1: In Vitro Mechanistic Validation, which must utilize human induced pluripotent stem cell (iPSC)-derived co-culture models (containing neurons, astrocytes, and microglia) from healthy donors and patients with genetic predispositions (e.g., familial AD mutations, *LRRK2* mutations for PD), as these systems provide a superior human-relevant context to immortalized rodent cell lines. The experiments in this tier involve several key steps: (1) Compound Standardization, conducting all experiments using highly purified (>98%) and structurally verified compounds; (2) Multi-Omic Profiling, performing unbiased, genome-wide screening (RNA-seq, miRNA-seq, circRNA-seq) to identify which ncRNAs are modulated; (3) Functional Causality Testing, using gain- and loss-of-function tools (e.g., miR-132 mimics/inhibitors, ASOs) to establish causality; and (4) Reporter Assays, to confirm direct molecular interactions (e.g., CREB-dependent transcription of miR-132).

This is followed by Tier 2: In Vivo Mechanistic and Efficacy Studies. This tier requires selecting animal models most appropriate for the hypothesis (e.g., the EAE model for remyelination) and adhering strictly to ARRIVE 2.0 guidelines [1,5,29,41,50,60,65,72]. Experiments include: (1) Cell-Type Specific Analysis, using advanced techniques like single-cell RNA-sequencing or TRAP-seq to understand changes in specific cell types in vivo; (2) Genetic Validation, using genetic models (e.g., conditional knockouts) to definitively test the necessity of an ncRNA; and (3) Dose–Response and PK/PD Correlation, to establish a clear pharmacokinetic/pharmacodynamic relationship.

Finally, Tier 3: Preclinical Translational Development focuses on addressing the critical hurdles that prevent promising compounds from reaching clinical trials. These studies include: (1) Formulation and Bioavailability, to develop optimized formulations for oral bioavailability and CNS penetration; (2) Target Engagement Biomarkers, investigating if specific ncRNAs can be detected in CSF or plasma-derived exosomes as non-invasive biomarkers; and (3) GLP Toxicology, conducting comprehensive safety studies under Good Laboratory Practice (GLP) to establish a safe dose range for first-in-human studies.

The proposed roadmap involves distinct technical and financial hurdles. Tier 1 (12–24 months) requires significant investment in high-throughput multi-omics and human iPSC technology to ensure human relevance. Tier 2 (2–4 years) faces the technical complexity of cell-type-specific in vivo analysis and the high cost of specialized transgenic models. Finally, Tier 3 (5+ years) represents the primary translational bottleneck, necessitating substantial funding for GLP-compliant toxicology and pharmacokinetic optimization to bridge the ‘valley of death’ between preclinical validation and clinical trials.

## 7. Conclusions

Despite these constraints, *Hericium erinaceus* bioactives—specifically mycelium-derived erinacines and fruiting body hericenones—possess substantial preclinical support for their neurotrophic potential. Data demonstrates their ability to stimulate NGF and BDNF synthesis, activate PI3K/AKT and MAPK/ERK cascades, and engage master regulators such as CREB and Nrf2. These activities translate to benefits in models of AD, PD, MS, stroke, and TBI.

This review proposes that ncRNAs represent the critical, unexplored layer connecting these bioactives to their downstream effects. We posit that these compounds orchestrate lasting changes in miRNAs, lncRNAs, and circRNAs by engaging key transcription factors. This framework advances traditional models by introducing a ‘stabilization layer’ that converts transient kinase signaling into enduring transcriptomic shifts, explaining sustained morphological changes like neurite extension.

Validating this hypothesis requires a paradigm shift toward rigorous, hypothesis-driven research using purified compounds, human iPSC-derived models, and multi-omics tools. Such an approach aligns with the shift toward network-based drug discovery, positioning *H. erinaceus* bioactives as multi-target modulators essential for treating the complex pathologies of neurodegeneration.

## Data Availability

No new data were created or analyzed in this study. Data sharing is not applicable to this article.

## Abbreviations

The following abbreviations are used in this manuscript:

3′ UTR	3′ untranslated region
Aβ	Amyloid-beta
AD	Alzheimer’s disease
ALS	Amyotrophic lateral sclerosis
AREs	Antioxidant Response Elements
BACE1	B-site APP cleaving enzyme 1
BACE1-AS	BACE1 natural antisense transcript
BBB	Blood–brain barrier
BDNF	Brain-Derived Neurotrophic Factor
CDR1as	Cerebellar Degeneration-Related protein 1 antisense
circRNAs	Circular RNAs
ciRS-7	Circular RNA sponge for miR-7
CNS	Central nervous system
CREB	cAMP response element-binding protein
ERK	Extracellular Signal-Regulated Kinase
GLP	Good Laboratory Practice
*H. erinaceus*	*Hericium erinaceus*
IDE	Insulin-degrading enzyme
iPSC	Induced pluripotent stem cell
lncRNAs	Long non-coding RNAs
MALAT1	Metastasis-Associated Lung Adenocarcinoma Transcript 1
MAPK	Mitogen-Activated Protein Kinase
miRNAs	MicroRNAs
mRNAs	Messenger RNAs
MS	Multiple Sclerosis
ncRNAs	Non-coding RNAs
NDPIH	N-de phenylethyl isohericerin
NEAT1	Nuclear Enriched Abundant Transcript 1
NGF	Nerve Growth Factor
OPCs	Oligodendrocyte precursor cells
PD	Parkinson’s disease
PI3K	Phosphoinositide 3-kinase
TBI	Traumatic brain injury
TrkA	Tropomyosin receptor kinase A
TrkB	Tropomyosin receptor kinase B
